# Intracardiac echocardiography guided zero-fluoroscopy workflow versus conventional fluoroscopy-guided workflow in atrial fibrillation ablation: efficiency, safety, and operator experience

**DOI:** 10.3389/fmed.2026.1928162

**Published:** 2026-09-11

**Authors:** Zhifu Yang, Hao Wu, Shanshan Zhuo, Lu Zhang, Ding Yuan, Shaopeng Wang, Lishan Lin, Yifan Xu, Xianfeng Du

**Affiliations:** 1 Department of Cardiology, Ningbo No. 2 Hospital, Wenzhou Medical University, Ningbo, Zhejiang, China; 2 Cardiovascular Medicine Center, Heyou Hospital, Foshan, Guangdong, China; 3 Health Science Center, Ningbo University, Ningbo, Zhejiang, China; 4 Procurement Department, Ningbo No. 2 Hospital, Wenzhou Medical University, Ningbo, Zhejiang, China

**Keywords:** atrial fibrillation, intracardiac echocardiography, lead apron protection, occupational fatigue, radiofrequency catheter ablation, zero-fluoroscopy ablation

## Abstract

**Background:**

Intracardiac echocardiography (ICE)-guided Zero-Fluoroscopy Workflow Group has been described in the literature. However, robust evidence simultaneously addressing procedural safety, efficiency, and operator experience remains limited. This study aims to systematically evaluate the procedural efficacy, intraoperative safety, and impact on operator experience of this technique.

**Objective:**

To compare the ICE-guided Zero-Fluoroscopy Workflow with Conventional fluoroscopy-guided workflow in the context of radiofrequency catheter ablation for atrial fibrillation (AF), and to quantify the effect of zero-fluoroscopy mode on operator workload and comfort.

**Methods:**

A retrospective study was conducted enrolling 177 patients who underwent radiofrequency catheter ablation for AF at Ningbo No. 2 Hospital between December, 2023 and June, 2026. Patients were divided into the ICE-Guided Zero-Fluoroscopy Workflow Group and the Conventional fluoroscopy-guided workflow Group. Operator subjective experience was assessed using the Visual Analogue Scale (VAS) for physical fatigue and procedural comfort immediately after each procedure. Total procedure time, TSP time, procedural success rate, and intraprocedural complications were also compared between groups.

**Results:**

Operator post-procedural VAS scores for physical fatigue (1.1 ± 0.6 vs. 6.8 ± 1.9) and procedural comfort (1.3 ± 0.7 vs. 7.2 ± 1.8) were significantly lower in the ICE-Guided Zero-Fluoroscopy Workflow Group compared to the Conventional fluoroscopy-guided workflow Group (both *P* < 0.001). Total procedure time, TSP time, and procedural success rate showed no statistically significant differences between groups (*P* > 0.05). One case of cardiac tamponade occurred in the Conventional fluoroscopy-guided workflow Group.while no intraoperative complications were observed in the ICE-guided group.

**Conclusion:**

In this retrospective single-center study, an ICE-guided zero-fluoroscopy workflow for AF ablation was feasible and demonstrated similar procedural efficiency to a conventional fluoroscopy-guided workflow. The zero-fluoroscopy strategy was not associated with an observed increase in acute complications in this cohort. In addition, it was associated with substantially lower operator-reported fatigue and discomfort, likely reflecting the absence of lead apron use. These findings support further prospective evaluation of ICE-guided zero-fluoroscopy workflows as a potentially valuable strategy for balancing procedural performance and operator occupational health.

## 1 Introduction

Catheter-based cardiac radiofrequency ablation represents the mainstream therapeutic strategy for patients with atrial fibrillation (AF) and is recommended by current clinical guidelines (1, 2). During the procedure, transseptal puncture (TSP) is a critical step, and the precision of puncture directly determines overall procedural safety. Traditional TSP relies primarily on fluoroscopic guidance, requiring operators to localize the fossa ovalis through indirect anatomical landmarks, such as the coronary sinus ostium and aortic root. However, conventional two-dimensional (2D) fluoroscopy cannot provide real-time assessment of the spatial relationship between the needle tip and the interatrial septum, raising the risk of serious complications, such as inadvertent cardiac perforation or aortic root puncture. Furthermore, fluoroscopy-guided procedures require operators to wear heavy lead protective equipment weighing 10–15 kg. Prolonged use of such equipment not only causes chronic musculoskeletal disorders (MSDs) affecting the cervical spine, lumbar spine, and musculoskeletal system (3–5), but also impairs the stability of delicate intraoperative manipulations due to physical fatigue. Meanwhile, both patients and medical staff face the additional risk of radiation exposure (6, 7).

In recent years, intracardiac echocardiography (ICE) has been widely popularized in interventional treatment of arrhythmias, allowing fully visualized and zero-fluoroscopy procedures (8–10). Although zero-fluoroscopy ablation guided by ICE has been progressively implemented in clinical practice, most existing studies have focused exclusively on objective patient-related outcomes, with limited quantitative assessment of operator occupational burden and procedural experience (11–15). The present retrospective study was designed to address these gaps.

## 2 Materials and methods

### 2.1 Study design

A retrospective study was conducted in 177 patients who underwent radiofrequency catheter ablation for AF at the Department of Cardiovascular Medicine, Ningbo No. 2 Hospital, from December 2023 to June 2026. Inclusion criteria: (1) age ≥18 but <85 years; (2) confirmed diagnosis of paroxysmal or persistent AF meeting indications for radiofrequency ablation. Exclusion criteria: (1) left atrial thrombus or severe valvular disease; (2) severe hepatic or renal insufficiency, or coagulation disorders; (3) inadequate ICE image quality precluding full-procedure monitoring. Patients were assigned to the ICE-Guided Zero-Fluoroscopy Workflow Group and Conventional fluoroscopy-guided workflow Group. The treatment allocation was non-randomized and determined based on a combination of patient choice (e.g., desiring a “green/zero-fluoroscopy” procedure), financial willingness to cover the additional cost of the ICE catheter, and the availability of the ICE equipment on the day of the procedure. Additionally, patients with complex anatomical features (such as a thickened interatrial septum, interatrial septal aneurysm, prior atrial septal defect closure, or a giant left atrium) identified via pre-procedural transesophageal echocardiography or left atrial CT were preferentially allocated to the ICE group.

To minimize potential selection and chronological biases, several measures were implemented. Firstly, both procedures were performed concurrently during the same study period (from December, 2023 to June, 2026), rather than using a historical cohort for the conventional group, thereby eliminating confounding factors related to temporal trends in perioperative management or ablation strategies. Secondly, to address the learning curve effect, all procedures were performed by the same experienced operator team who had completed a one-year specialized fellowship in green electrophysiology at the Sir Run Run Shaw Hospital affiliated with Zhejiang University School of Medicine, suggesting that the operators had substantial prior experience before the study period.

**Figure 1:**
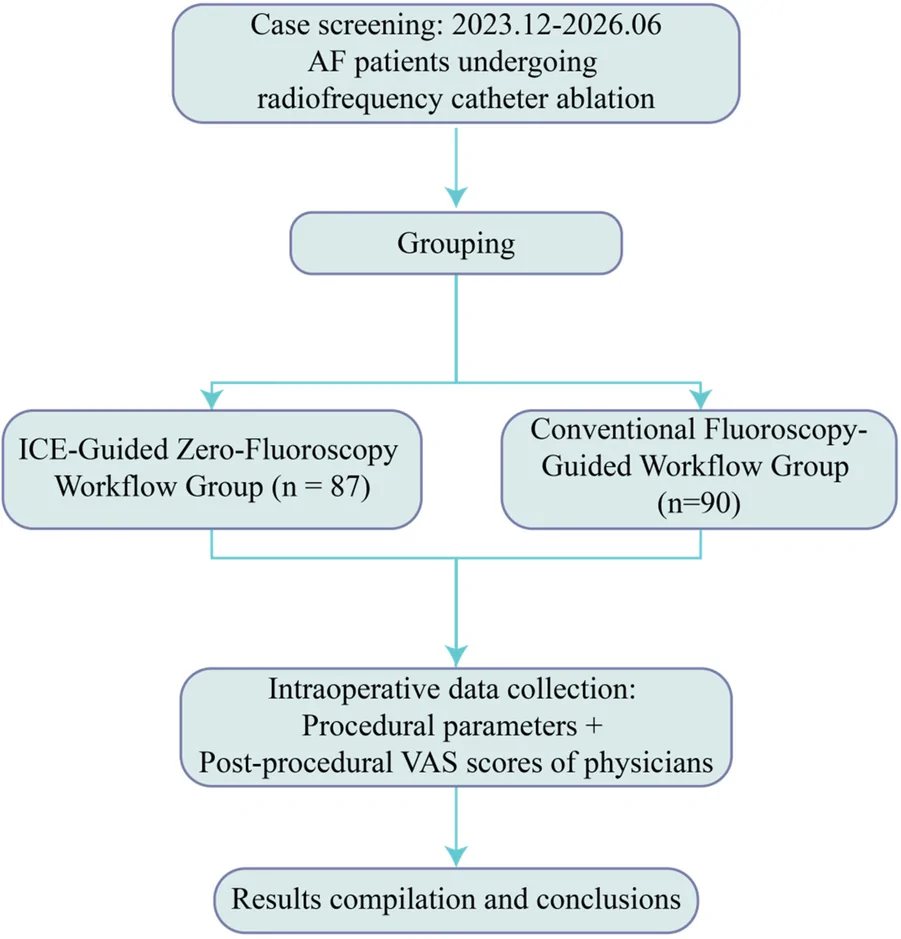
Flow sheet of the study.

To minimize potential temporal and learning-curve biases, both workflows were performed during the same study period by the same experienced electrophysiology team. Because treatment allocation was non-randomized, residual selection bias cannot be excluded. This study was approved by the Medical Ethics Committee of Ningbo No. 2 Hospital (Figure 1).

### 2.2 Procedural methods

All patients underwent ablation using the CARTO® 3 three-dimensional electroanatomical mapping system (Biosense Webster). Pre-procedural transesophageal echocardiography (TEE) or left atrial CT angiography (CTA) was performed 24–48 h prior to the operation to rule out thrombi within the left atrial appendage and left atrium. Procedures were conducted under local anesthesia with conscious sedation, and heparin was administered intraoperatively to maintain activated clotting time (ACT) between 250 and 350 s. In this study, patients with paroxysmal atrial fibrillation (PAF) underwent bilateral pulmonary vein isolation (PVI) alone. For those with persistent atrial fibrillation (PeAF), additional linear ablations at the left atrial roof and inferior wall were performed following PVI to create a BOX lesion for posterior wall isolation, and substrate modification was applied to low-voltage zones if necessary.

### 2.3 ICE-guided zero-fluoroscopy workflow group

**Figure 2:**
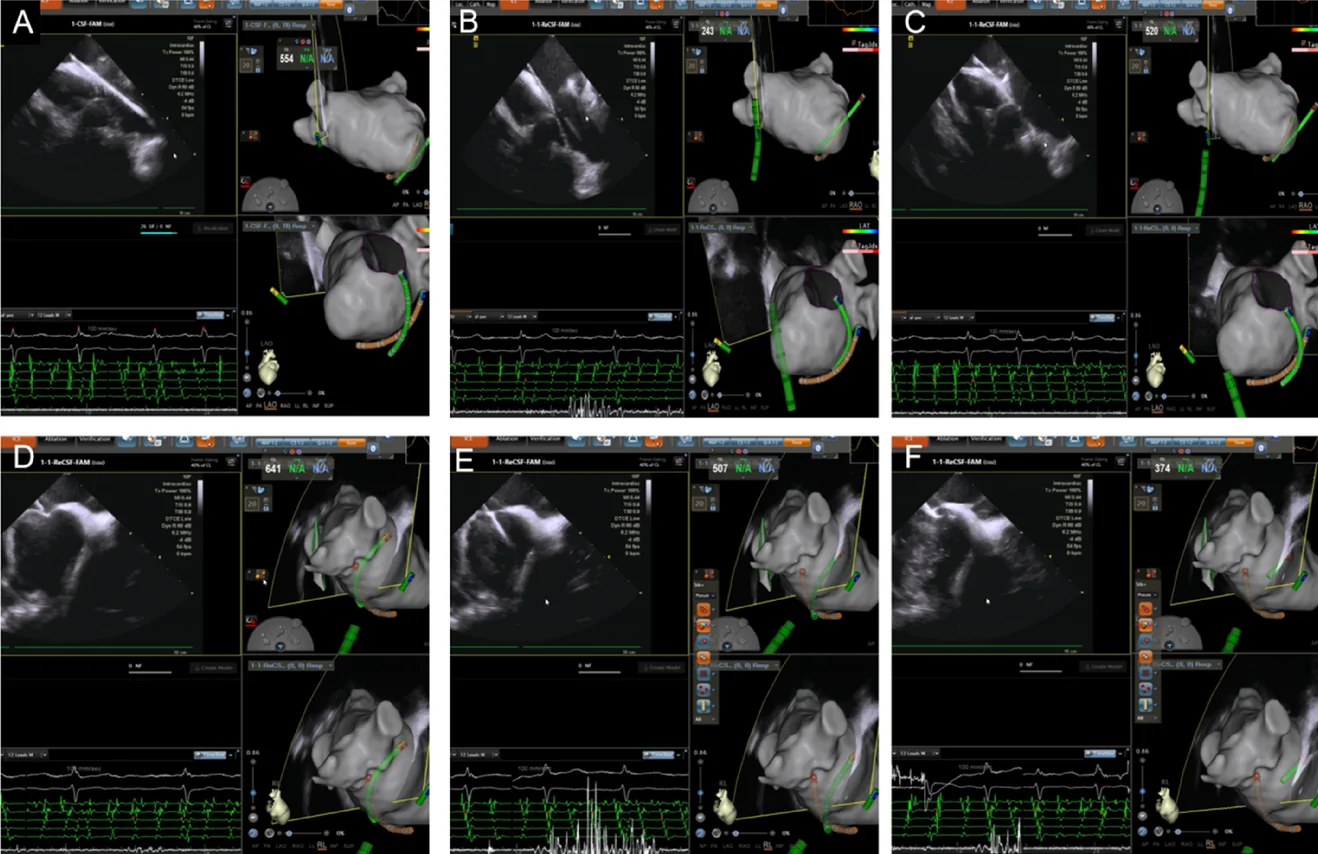
Method of electrified J-wire- assisted transseptal puncture under ICE guidance. Clarification of Figure 2: (**A**) 0.032-inch guidewire was advanced into the superior vena cava under ICE monitoring. (**B**) The steerable visualized sheath was advanced to the superior vena cava under ICE and three-dimensional mapping guidance. (**C**) ICE real-time tracking of the sheath position was performed by tracing the fan plane within the steerable visualized sheath. (**D**) After identifying the optimal puncture site by ICE, the steerable visualized sheath was pushed against the interatrial septumto create a maximal “tenting” sign. (**E**) Atrial septal puncture was performed using the electrocautery mode of the Electrified J- Wire. Immediate breakthrough was observed, accompanied by the appearance of vaporization “white smoke” on the ICE fan plane. (**F**) The 0.032-inch guidewire was then redirected downward and advanced into the left superior pulmonary vein.

Following standard disinfection and femoral venous access, an ICE catheter (SoundStar, Biosense Webster) was advanced to the right atrium to exclude left atrial appendage/left atrial thrombus and pericardial effusion. Under ICE guidance, a 0.032-inch J-wire was advanced to the superior vena cava (SVC), followed by an adjustable-curve sheath (Vizigo, Biosense Webster) advanced along the wire to the SVC. The Vizigo sheath was then steered to the fossa ovalis under ICE guidance, and the optimal puncture site was confirmed by generating a maximal “convertible roof sign” on the interatrial septum. The J-wire was gently withdrawn until its tip protruded approximately 1 mm beyond the dilator tip. An electrosurgical unit set to 40 W was applied to the proximal segment of the J-wire to deliver energy for TSP. The appearance of “white smoke” (microbubbles) in the left atrium on ICE imaging served as the immediate signal to cease energy delivery. The J-wire was then advanced to the left superior pulmonary vein, and the sheath was advanced over the wire into the left atrium. The procedure was performed under zero fluoroscopy, and no lead aprons were required for the operator or assisting staff (Figure 2).

### 2.4 Conventional fluoroscopy-guided workflow group

**Figure 3:**
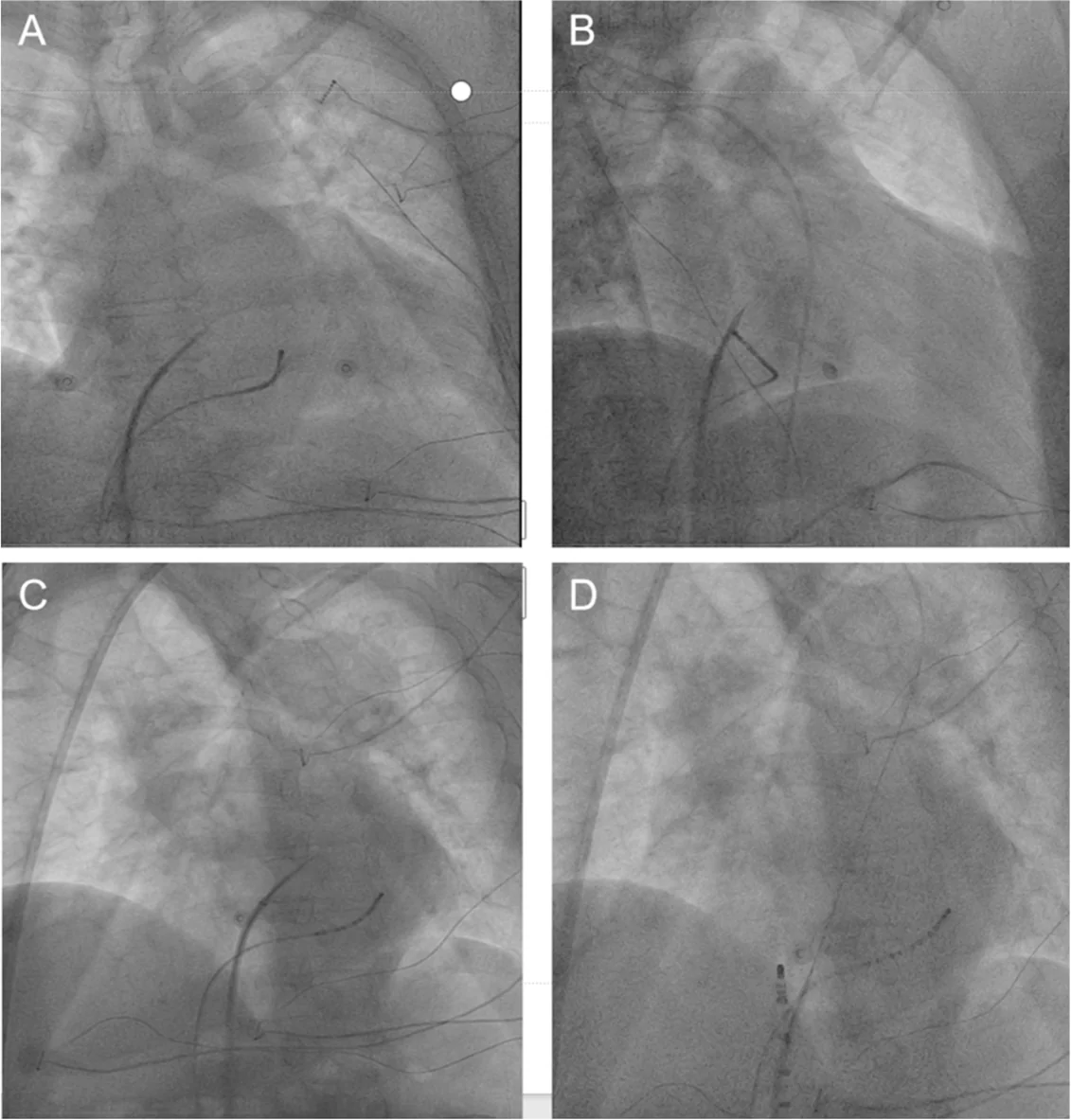
Method of atrial septal puncture under fluoroscopic guidance. Clarification of Figure 3: **(A)** Three “jumps” were observed during withdrawal of the fixed-curve sheath to the fossa ovalis under fluoroscopy. **(B)** Transseptal puncture was performed in the right anterior oblique (RAO) 45° view. **(C)** Successful puncture was confirmed by the diffuse spread of contrast medium in the left atrium under fluoroscopy. **(D)** Under fluoroscopic guidance, the guidewire was advanced through the puncture sheath into the left superior pulmonary vein (LSPV), where the “double-track” sign was observed.

Following standard disinfection and femoral venous access, a Swartz sheath (SL1; Abbott) was advanced along a J-wire to the SVC. The fossa ovalis was localized under fluoroscopy (RAO 45°). TSP was performed using a transseptal needle with fluoroscopic confirmation of needle tip position and puncture depth. The J-wire was then advanced to the left superior pulmonary vein, and the sheath was advanced into the left atrium. The entire procedure required full lead apron protective equipment for all operating staff (Figure 3).

### 2.5 AI-guided radiofrequency catheter ablation

Both groups underwent radiofrequency catheter ablation using an ST catheter under AI (ablation index) guidance, with target AI values of 500 for the anterior wall, 400 for the posterior wall, 450 for the inferior wall, 470 for the superior wall, and 500 for the ridge. Power was set at 40 W, and the saline irrigation flow rate was 30 mL/min during radiofrequency ablation. During the ablation process, catheter contact force and local impedance were monitored in real time. The contact force was targeted at 10–20 g to ensure optimal lesion formation while maintaining safety. An instantaneous local impedance drop of ≥10 *Ω* was used as an adjunctive marker of lesion formation. For patients in the ICE group, ICE was continuously performed throughout the procedure to monitor for potential peri-procedural complications, particularly pericardial effusion.

### 2.6 Post-procedural anticoagulation

All patients received oral anticoagulation for at least 3 months following the procedure. Continuation beyond 3 months was determined by individual stroke risk assessment using the CHA₂DS₂-VASc score.

### 2.7 Study endpoints

Total procedure time was defined as the interval from groin puncture to removal of all venous sheaths. Total TSP time was defined as the interval from initiation of SVC pullback to successful advancement of the sheath into the left atrium. Immediately following each procedure, two subjective VAS scores (0–10) were recorded for the primary operator: (1) physical fatigue VAS score (higher scores indicating greater fatigue of the neck, shoulder, and lower back); and (2) procedural discomfort VAS score (higher scores indicating greater discomfort). Scores in the Conventional fluoroscopy-guided workflow Group. were obtained after operators wore full lead aprons, whereas scores in the ICE-Guided Zero-Fluoroscopy Workflow Group were obtained without lead apron use. Primary safety endpoint: TSP-related serious complications, including inadvertent aortic puncture, new pericardial effusion, symptomatic stroke or systemic embolism, and death. Primary efficacy endpoints: TSP time and total procedure time.

## 3 Statistical analysis

Statistical analyses were performed using SPSS version 29.0. Normally distributed continuous variables are presented as mean ± standard deviation, and non-normally distributed variables as median (interquartile range). Intergroup comparisons were performed using the Student's t-test (normal distribution) or the Mann–Whitney U test (non-normal distribution). Categorical variables were expressed as counts and percentages, and compared using the chi-squared test or Fisher's exact test. Statistical significance was defined as two-sided *P* < 0.05.

## 4 Results

### 4.1 Baseline characteristics

A total of 177 patients were retrospectively enrolled. The ICE-Guided Zero-Fluoroscopy Workflow Group comprised 87 patients (mean age 65.97 ± 9.3 years, 55% male, 41% paroxysmal AF), and the Conventional fluoroscopy-guided workflow Group. comprised 90 patients (mean age 65.07 ± 8.8 years, 64% male, 43% paroxysmal AF). No statistically significant differences were observed in most baseline characteristics, except for a higher prevalence of heart failure in the conventional fluoroscopy-guided workflow group (*P* = 0.028) (Table 1).

**Table 1:** Baseline characteristics.

Variables	ICE-Guided Zero-Fluoroscopy Workflow Group (*N* = 87)	Conventional fluoroscopy-guided workflow Group (*N* = 90)	*P* value
Age (years)	65.97 ± 9.31	65.19 ± 8.73	0.578
Male (%)	48 (55.2%)	58 (64.4%)	0.208
BMI(kg/m^2^)	24.79 ± 3.58	23.95 ± 3.26	0.127
Smoking (%)	14 (16.1%)	23 (25.6%)	0.122
Alcohol (%)	11 (12.6%)	19 (21.1%)	0.133
Paroxysmal AF (%)	41 (47.1%)	43 (47.8%)	0.931
Coronary artery disease (%)	7 (8.0%)	9 (10.0%)	0.65
Hypertension (%)	46 (52.9%)	41 (45.6%)	0.33
Diabetes mellitus (%)	15 (17.4%)	15 (16.7%)	0.891
Heart failure (%)	8 (9.2%)	19 (21.1%)	0.028
LAD (mm)	42.03 ± 5.80	42.14 ± 4.68	0.789
LVEF (%)	62.67 ± 6.81	62.83 ± 8.54	0.381

AF, atrial fibrillation; ICE, intracardiac echocardiography; LAD, left atrial diameter; LVEF, left ventricular ejection fraction; TSP, transseptal puncture.

### 4.2 Procedural details

All patients in both groups successfully completed TSP. TSP time, total procedure time, and procedural success rate showed no statistically significant differences between the ICE-Guided Zero-Fluoroscopy Workflow Group and the Conventional fluoroscopy-guided workflow Group.(*P* > 0.05) (Table 2). Although one case of cardiac tamponade occurred in the Conventional fluoroscopy-guided workflow Group, whereas no intraoperative complications were observed in the ICE-guided TSP group throughout the observation period, suggesting a potential safety advantage of ICE guidance. However, this finding requires validation in larger-scale studies.

**Table 2:** Procedural outcomes.

Outcome	ICE-Guided Zero-Fluoroscopy Workflow Group (*N* = 87)	Conventional fluoroscopy-guided workflow Group (*N* = 90)	*P* value
Procedure time (min)	119.20 ± 35.28	127.51 ± 30.52	0.098
Total TSP time (s)	178.68 ± 66.04	198.80 ± 66.14	0.077
Puncture success rate	98.90%	100%	0.492
PVI alone, *n* (%)	41 (47.1%)	43 (47.8%)	0.931
PVI + Additional Ablation	46 (52.9%)	47 (52.2%)	0.931

### 4.3 Post-procedural VAS scores

Operator post-procedural VAS scores for physical fatigue (1.1 ± 0.6 vs. 6.8 ± 1.9) and procedural comfort (1.3 ± 0.7 vs. 7.2 ± 1.8) were both significantly lower in the ICE-Guided Zero-Fluoroscopy Workflow Group compared to the Conventional fluoroscopy-guided workflow Group. (both *P* < 0.001). Operators in the ICE-Guided Zero-Fluoroscopy Workflow Group performed procedures without lead aprons throughout; accordingly, both post-procedural physical fatigue and procedural comfort scores were significantly lower than those in the lead-apron-wearing Conventional fluoroscopy-guided workflow Group. (*P* < 0.001) (Table 3).

**Table 3:** Post-procedural VAS scores.

Outcome	ICE-Guided Zero-Fluoroscopy Workflow Group (*N* = 87)	Conventional fluoroscopy-guided workflow Group (*N* = 90)	*P* value
Fatigue score	1.09 ± 0.64	6.83 ± 1.94	<0.0001
Comfort score	1.34 ± 0.73	7.18 ± 1.78	<0.0001

## 5 Discussion

To the best of our knowledge, this study provides a quantitative comparison of an ICE-guided zero-fluoroscopy workflow and a conventional fluoroscopy-guided workflow for AF ablation, with attention to procedural efficiency, acute safety, and operator-reported experience. The principal findings were as follows: first, the ICE-guided zero-fluoroscopy workflow was feasible and achieved similar procedure time, transseptal puncture time, and acute success rate compared with the conventional workflow. Second, no acute TSP-related complications were observed in the ICE-guided group, whereas one case of cardiac tamponade occurred in the conventional group; however, given the small number of events and the retrospective design, this finding should be interpreted cautiously and cannot establish comparative safety. Third, operator-reported fatigue and discomfort were substantially lower in the zero-fluoroscopy workflow group, suggesting a potentially improved procedural experience.

### 5.1 Efficacy and device optimization

In the present study, TSP time and success rate did not differ significantly between groups (*P* > 0.05), suggesting that ICE-Guided Zero-Fluoroscopy Workflow Group achieved clinical reliability and efficiency similar to those of conventional approaches. From a technical standpoint, ICE may transform TSP from a fluoroscopy-dependent procedure into a procedure guided by direct ultrasound visualization. With the conventional BRK needle technique, unsuccessful initial attempts necessitate a repetitive cycle of needle withdrawal, re-advancement of the J-wire to the SVC, and needle exchange—a cumbersome process that is both time-consuming and associated with potential risk. In contrast, the electrified J-wire technique achieves precise initial positioning at the optimal puncture site, substantially reducing futile attempts caused by positional deviation.

Moreover, for challenging anatomical scenarios such as thickened, fibrotic, or aneurysmal interatrial septa, or in patients with prior atrial septal occluder devices, the BRK needle relying solely on mechanical force is prone to slippage or displacement. The electrified J-wire technique utilizes high-frequency electrical energy (40 W in the present study) to instantaneously penetrate the septum with minimal mechanical force, while real-time ICE imaging provides simultaneous resolution of adjacent anatomical structures, effectively preventing inadvertent injury to the posterior left atrial wall or pericardium that can result from uncontrolled forward advancement of a conventional needle at the moment of septal breakthrough (16–20).

### 5.2 Safety and ICE guidance

TSP is among the highest-risk maneuvers in AF ablation. Conventional fluoroscopy provides only 2D projections, and operators must rely on indirect signs—such as the “jump sign”—to infer needle tip position, with inherent risk of inadvertent puncture of the aortic root or pericardium. In the present study, one case of cardiac tamponade occurred in the Conventional fluoroscopy-guided workflow Group, whereas no complications were observed in the ICE-guided group.

The incorporation of ICE converts “blind puncture” into fully visualized, real-time direct-view operation. Under continuous ICE monitoring, the operator can clearly visualize the maximal tenting sign produced by the Vizigo sheath at the fossa ovalis and continuously track the spatial relationship between the guidewire tip and adjacent structures, including the aorta and posterior left atrial wall. Furthermore, the appearance of a “white smoke” vapor sign (microbubble sign) within the ICE imaging field serves as an immediate and sensitive indicator of successful septal crossing, prompting the operator to immediately cease energy delivery, thereby minimizing the risk of thermal injury and systemic embolism from tissue charring (21–25). Notably, the clinical benefits in the ICE group stem from the synergistic workflow—integrating real-time ICE imaging, a steerable sheath, and an electrosurgical guidewire under 3D mapping—rather than any single device. Additionally, the ablation strategy (PVI alone vs. PVI with linear lesions) did not confound our outcomes, The real-time visualization of ICE optimized catheter-tissue contact and positioning during linear ablation, effectively offsetting the potential procedural prolongation and safety risks associated with more extensive ablation.

### 5.3 Green electrophysiology and occupational health

With the rapidly increasing volume of AF ablation procedures, occupational health concerns among interventional cardiologists have received growing attention. Prolonged use of lead aprons weighing 10–15 kg is a primary contributor to cervical spondylosis, lumbar disc disease, and MSDs in interventional physicians (26–30). The present study, aligned with the international trend toward “Green Electrophysiology” and “zero-fluoroscopy ablation,” We used pragmatic VAS scores to capture operator-reported fatigue and comfort as exploratory measures of procedural experience. Results demonstrated that physical fatigue and comfort scores were significantly lower in the ICE-guided group compared to the control group (*P* < 0.001). Under full ICE navigation, operators were able to perform precise catheter manipulation without lead apron encumbrance. This substantially reduced occupational physical fatigue, extended the professional longevity of interventional cardiologists, and helped maintain hand stability during prolonged, high-intensity procedures—indirectly contributing to improved procedural precision and safety (31–35).

## 6 Limitations

This study has several limitations. First, as a single-center, retrospective, non-randomized study, patient allocation was subject to selection bias related to clinical preferences and financial factors. Although concurrent enrollment and a single experienced electrophysiology team helped minimize temporal and learning-curve biases, prospective randomized controlled trials are still warranted. Second, because we compared integrated workflows rather than isolated components, the individual contributions of the electrosurgical J-tipped guidewire and ICE guidance could not be independently quantified. Since ICE is rarely paired with conventional BRK needles in our practice, future prospective, three-arm randomized trials (comparing fluoroscopy-guided BRK, ICE-guided BRK, and ICE-guided electrosurgical-wire approaches) are warranted to dissect these effects. Third, this study was not designed as a formal non-inferiority trial. Lacking a pre-specified non-inferiority margin (*Δ*), formal sample size calculations, and confidence interval criteria, the non-significant differences (*P* > 0.05) in efficiency and safety suggest descriptive comparability rather than confirmatory statistical equivalence. Future prospective trials with rigorous statistical powering are required for validation. Fourth, this study primarily focused on acute intraoperative safety and procedural feasibility; thus, long-term arrhythmia recurrence was not designated as a primary endpoint. Although a standardized follow-up protocol (including 12-lead ECG and 24-hour Holter monitoring at 1, 3, 6, and 12 months) was implemented, the median follow-up duration was relatively short at 7.2 months (IQR: 3.5–11.6 months), with a 4.0% loss-to-follow-up rate. Because patient enrollment concluded recently, a portion of the cohort had not yet reached the 12-month post-procedural milestone at the time of data analysis. Consequently, robust long-term efficacy comparisons between the two workflows remain to be established in future studies with extended follow-up periods. Five,the VAS scores were subjective, unblinded, and potentially correlated within operators across repeated procedures; therefore, they should be interpreted as exploratory measures.

## 7 Conclusion

The ICE-guided zero-fluoroscopy workflow group achieved procedural efficiency similar to that of the conventional fluoroscopy-guided workflow group in terms of procedure time, TSP time, and success rate, without prolonging the procedural workflow. In addition, intraoperative safety is favorable. Notably, compared to Conventional fluoroscopy-guided workflow Group, this technique significantly reduces operator post-procedural physical fatigue and procedural discomfort by enabling complete zero-fluoroscopy operation and obviating lead apron wear. This approach represents a viable alternative for AF radiofrequency catheter ablation that simultaneously balances procedural efficiency, patient safety, and operator occupational health.

## Data Availability

The original contributions presented in the study are included in the article/Supplementary Material, further inquiries can be directed to the corresponding author.
